# Satellite Tracking of Manta Rays Highlights Challenges to Their Conservation

**DOI:** 10.1371/journal.pone.0036834

**Published:** 2012-05-10

**Authors:** Rachel T. Graham, Matthew J. Witt, Dan W. Castellanos, Francisco Remolina, Sara Maxwell, Brendan J. Godley, Lucy A. Hawkes

**Affiliations:** 1 Wildlife Conservation Society, Gulf and Caribbean Sharks and Rays Program, Punta Gorda, Belize; 2 Centre for Ecology and Conservation, University of Exeter, Tremough Campus, Penryn, Cornwall, United Kingdom; 3 Environmental and Sustainability Institute, University of Exeter, Tremough Campus, Penryn, Cornwall, United Kingdom; 4 Wildlife Conservation Society, Punta Gorda, Belize; 5 Reserva de la Biosfera Tiburón Ballena y Parque Nacional Isla Contoy, Domino Project, National Commission of Protected Areas, Cancun, Quintana Roo, Mexico; 6 Marine Conservation Institute, Glen Ellen California, United States of America; 7 Long Marine Laboratory, University of California Santa Cruz, Santa Cruz, California, United States of America; 8 Brambell Laboratories, Bangor University, Bangor, Gwynedd, United Kingdom; Institute of Marine Research, Norway

## Abstract

We describe the real-time movements of the last of the marine mega-vertebrate taxa to be satellite tracked – the giant manta ray (or devil fish, *Manta birostris*), the world's largest ray at over 6 m disc width. Almost nothing is known about manta ray movements and their environmental preferences, making them one of the least understood of the marine mega-vertebrates. Red listed by the International Union for the Conservation of Nature as ‘Vulnerable’ to extinction, manta rays are known to be subject to direct and incidental capture and some populations are declining. Satellite-tracked manta rays associated with seasonal upwelling events and thermal fronts off the Yucatan peninsula, Mexico, and made short-range shuttling movements, foraging along and between them. The majority of locations were received from waters shallower than 50 m deep, representing thermally dynamic and productive waters. Manta rays remained in the Mexican Exclusive Economic Zone for the duration of tracking but only 12% of tracking locations were received from within Marine Protected Areas (MPAs). Our results on the spatio-temporal distribution of these enigmatic rays highlight opportunities and challenges to management efforts.

## Introduction

Satellite tracking has yielded key information about the life history of marine vertebrates, many of which engage in long migrations (travelling thousands of kilometres) [1] and make deep dives [2], beyond the temporal and logistical abilities of researchers to follow them. The insights afforded by such tracking have provided structure around which conservation frameworks and regulations can be built [3] and an understanding of spatial ecology around which marine protected areas (MPAs) can be established (e.g. [4]). Satellite tracking has further provided parameters for models of distribution to enable forecasting of effects of, e.g. climate change, to marine vertebrates (e.g. [5], [6]).

Manta rays (or devil fish, *Manta birostris*) are the world's largest batoid fish (reaching a measured disc width of 7.1 m), with slow growth and low fecundity, birthing only one or two live ‘pups’ every one to two years following a gestation period of 12 months [7]. They are listed by the International Union for Conservation of Nature (IUCN) as “Vulnerable” to extinction [7] and included on Appendix I and II of the Convention on Migratory Species of Wild Animals. Recently Manta rays were found to encompass a second species *Manta alfredi* that ranges throughout the Central Eastern Atlantic and Indo-Pacific and possibly a third species constrained to the Gulf of Mexico and the Caribbean [8]. They are known to be purposefully and accidentally captured in fisheries operations and populations in the Pacific, Indian Ocean and Caribbean are apparently declining [7]. Critical information for conservation planning, such as knowledge on their movements and ecology, are however lacking. Indeed, the manta rays may be the least understood of the marine mega-vertebrate groups, and one of the last to be satellite tracked.

Manta rays are most often reported in coastal areas and continental shelves, near seamounts and in upwelling zones [9], [10], [11]. From unpublished reports and popular media, it would appear that manta rays are known to congregate in enormous numbers (up to hundreds of individuals) in some areas (e.g. Mexico, Mozambique, Maldives, Hawaii and Micronesia) for courtship, breeding and to visit cleaning stations. While manta rays are thought to remain resident to some areas [7], particularly the smaller and more coastally-constrained *M. alfredi* [10], in other areas they are thought to make seasonal long-distance migrations away from breeding areas, although non-breeding sites are not known [12].

Here, we describe the use of real-time satellite telemetry to gather insights into manta ray movements, allowing us to begin to generate environmental parameters for their distribution and assess the extent to which manta rays occur in protected areas.

**Figure 1:**
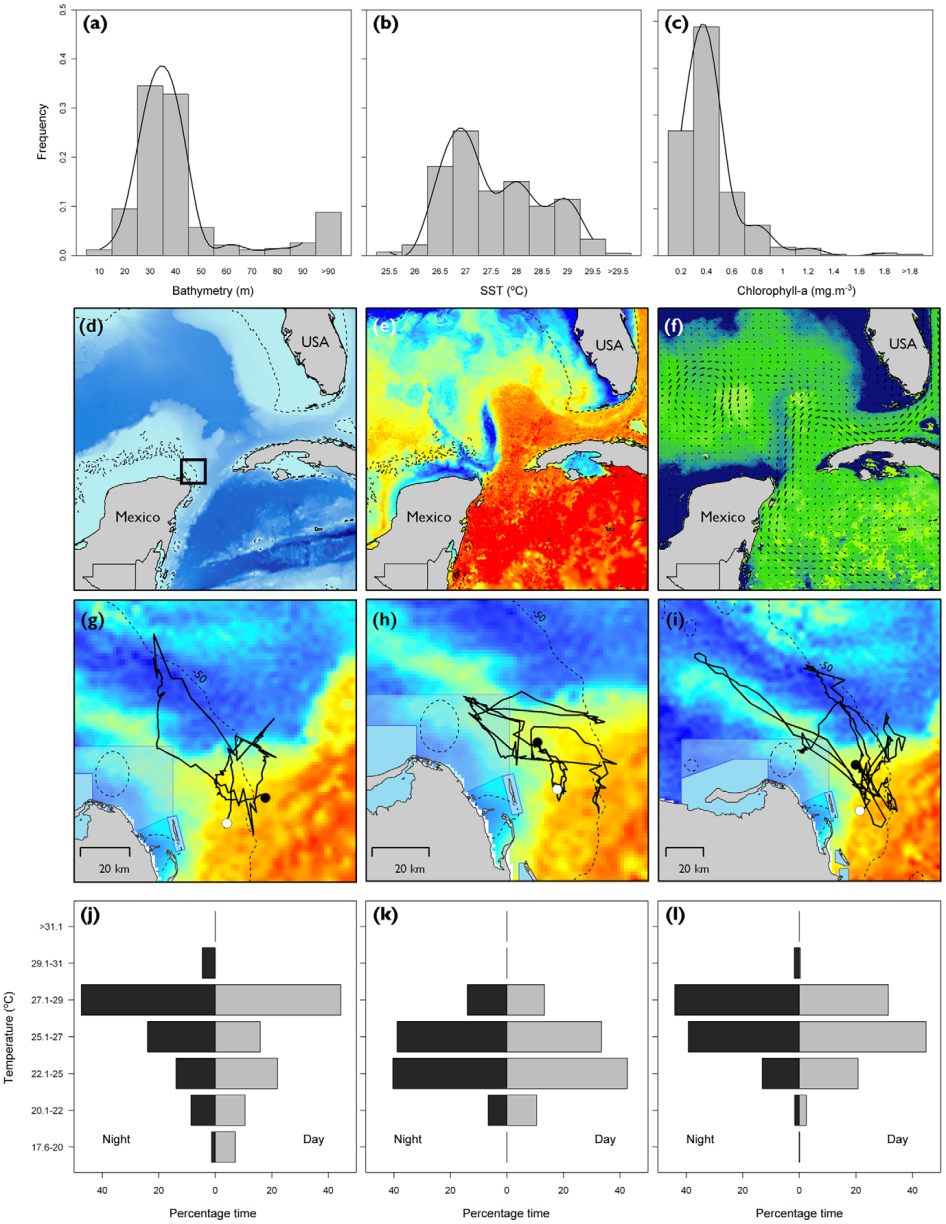
Movements of manta rays in the western Caribbean and south-east Gulf of Mexico. Frequency histograms of (a) bathymetry, (b) SST and (c) chlorophyll-α determined from the locations of all satellite tracked manta rays. Regional mapping of (d) bathymetry, (e) SST and (f) Chlorophyll-α imagery with geostrophic currents for 10^th^ Oct 2010. (g–i) Tracks of three of the six manta rays (one female, one male and the juvenile manta ray, mantas 1, 5 and 6, Table 1) are shown with SST imagery (10^th^ October). (j–l) Mean percentage time at temperature plots during night and day (temperature recorded by animal-borne tags) for the same individuals.

**Figure 2:**
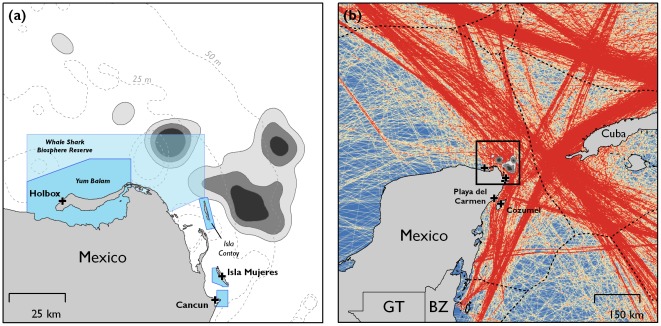
Utilisation distribution of manta ray locations (a) (quartic kernelling; grey polygons showing 25%, 50%, 75%, from darkest to lightest grey). Blue polygons show marine protected areas, tourism ports are indicated (black crosses). Commercial shipping activity, showing transit of boats belonging to the World Meteorological Organisation Voluntary Observing Ship Scheme (b) (red showing higher density of ship transit) from [41]. Core manta ray foraging areas are indicated, with Mexican tourism ports (Holbox, Isla Mujeres, Cancun, Playa del Carmen and Cozumel).

## Materials and Methods

We deployed six towed satellite transmitting position-only tags (Wildlife Computers SPOT5; http://wildlifecomputers.com/spot.aspx) on manta rays, in the southern Gulf of Mexico near Mexico's Yucatan peninsula over the duration of a 13-day research cruise. The research was carried out under permit from the Mexican federal government agency (OFICIO NÚM/SGPA/DGVS/05241). Tags were programmed to record ambient temperature (in asynchronous binning intervals selected to maximise recording around crespuscular periods, at 00:00, 05:00, 11:00, 12:00, 17:00 and 23:00) and to transmit continuously at the sea surface. The tags' position was determined by the Argos System (www.argos-system.org). Tags were attached while swimming behind and above the animal, using a small percutaneous nylon umbrella dart attached to a 1 m long 1/16″ (1.59 mm) stainless steel cable containing a mid-line swivel, inserted into the lower left or lower right quadrant shoulder musculature using a 2 m pole spear. The tags were covered with dark blue antifouling paint to minimize bio-fouling. Manta ray body size, or disc width, the distance between the two unfurled wingtips ±50 cm, was estimated by comparing the ray to a 2 m tagging pole or a snorkeler of known height. Sex was determined by the presence of ‘claspers’ (male sexual organs) [13]. Despite their size, manta rays are cryptic and rarely encountered, we thus applied tags to all the manta rays we were able to encounter during the 13-day sampling period.

Tag-derived ambient temperature data were expressed as a proportion of time spent within predetermined temperature ranges by local night and day periods. These were calculated using the NOAA sunrise/sunset calculator (http://www.srrb.noaa.gov/highlights/sunrise/calcdetails.html) with manta ray latitudes and longitudes, custom coded into MATLAB.

Argos data was filtered to only include location classes (LC) A, B, 0, 1, 2 and 3 for which location accuracy has been determined [14], [15]; locations with LC Z were removed. Unrealistic locations were also removed (swimming speeds greater than 20 km/hr). A behaviourally switching state-space model (SSM) was applied to Argos tracking data to handle observation error, improve data retention, and infer animal behavioural state (referred to as ‘transiting’ and ‘foraging’) from the movement patterns [16]. We used the model originally described by Jonsen et al [17] and refined by Breed et al [16], which has been successfully applied to a number of marine species including pinnipeds [16], [18], sea turtles [19], [20], [21] and cetaceans [22]. The SSM was generated using the software packages R and WinBUGS, and we estimated locations at five-hour intervals, reflecting the average number of Argos locations we received per day [16]. Model parameters were estimated using Markov Chain Monte Carlo (MCMC) estimation from two MCMC chains. We used 10,000 iterations after a burn-in of 5,000 and thinned by five to give the mean and variance for each location and behavioural parameter. Behaviour was discriminated into the two states based on the mean turning angle (γ) and autocorrelation in speed and direction (θ). We observed a lack of overlap between the parameters representing the opposing behavioural states, which indicated a true differentiation in movement patterns.

Movement metrics, describing transit speed and distance travelled, and coincident environmental data (sea surface temperature, chlorophyll-α, bathymetry and sea surface currents) were determined for each position. Environmental data were downloaded from the GODAE High Resolution Sea Surface Temperature Pilot Project (SST, http://ghrsst-pp.metoffice.com/, at ∼1 km resolution), NASA Goddard space flight centre Ocean colour (chlorophyll-α, http://oceancolor.gsfc.nasa.gov/SeaWiFS/,∼4 km product), the General Bathymetric Chart of the Oceans, GEBCO (bathymetry, http://www.gebco.net/, at 30 seconds arc resolution) and CLS AVISO OceanObs (sea surface currents, http://www.aviso.oceanobs.com/, at a resolution of 0.3° at the equator). Locations were also overlaid with the World Database of Protected Areas (http://www.unep-wcmc.org/wdpa) to assess the proportion of locations that were received from within Marine Protected Areas (MPAs).

Areas of high use by manta rays were determined using a quartic kernelling approach [23]. Data were first resolved to the best daily location per individual (if >1 highest location classes were received, the earliest was used) and data from all individuals was grouped for analysis. A utilisation distribution was subsequently created from the satellite tracking data using a smoothing parameter, *h*, of 10 km (which best represented the underlying spatial architecture of the location data) on a 1×1 km grid and percent volume contours (25, 50 and 75%) were created from the resulting raster.

**Table 1:** Deployment metrics for six manta rays.

Manta Ray	Location	Deployed	Detached	Duration	Sex	DW (cm)
1	Oligotrophic	10-Sep-10	10-Oct-10	32	F	400
2	Eutrophic	20-Jul-10	10-Aug-10	19	F	300
3	Eutrophic	21-Jul-10	24-Jul-10	4	F	450
4	Oligotrophic	08-Sep-10	10-Sep-10	2	F	400
5	Oligotrophic	09-Sep-10	14-Oct-10	41	UNK	400
6	Oligotrophic	08-Sep-10	29-Oct-10	64	M	350
Mean (range)				27 (2–64)		383 (350–450)

## Results

Tags provided data for a mean of 27 days (±21.6 s.d., range 2 to 64 days, Table 1). Tagged animals (n = four females, one male and one juvenile ray of indeterminate sex) remained in frontal zones off the Yucatan peninsula, traversing them repeatedly. Tracks showed strong separation between the state-space model behavioural parameters (γ and θ). Manta rays were in ‘foraging’ state for 97.7% of the locations received from the Argos System, moving at 1.2 km.h^−1^ (grand median of medians per individual, range 0.9 to 1.7 km.h^−1^) with animals covering as much 1,151 km before transmission ceased (Fig. 1, cumulative straight line distance between locations, mean track length 368±425 km s.d.). Manta rays moved up to 116 km away from their tag attachment locations and remained within Mexico's territorial jurisdiction for the duration of tracking. Most manta ray locations occurred further than 20 km offshore (92% of all locations) and only 11.5% locations occurred within MPAs (Fig. 2a). Areas with high relative densities of manta ray locations overlapped with dominant shipping routes within the region (Fig. 2b). There were no apparent differences in movement patterns by sex or body size (Table 1), or with ambient water-column temperature (Fig. 1j–l).

Satellite-tracked manta rays were rarely located in water deeper than 50 m (83% of all locations from waters shallower than 50 m, with 92% of all locations received from waters between 5 and 100 m deep, Fig. 1). Manta rays foraged in waters with sea surface temperatures ranging from 25.1 to 30.0**°**C, with 95% of all locations occurring in waters warmer than 26.1**°**C. The majority of manta ray locations occurred in waters with surface chlorophyll-α values between 0.14 and 0.76 mg.m^−3^ (5^th^ to 95^th^ percentiles, median 0.28 mg.m^−3^), and geostrophic current speeds of 8.4 to 94.0 cm.sec^−1^ (5^th^ to 95^th^ percentiles, median 76.6 cm.sec^−1^).

During our 13 days of boat surveying, including the period in which satellite tags were deployed, we made opportunistic plankton tows (using a 212 µm mesh, 50 cm diameter net) when we observed manta rays ram filter feeding at the surface and sub-surface to identify the prey species they were consuming. Manta rays were observed feeding in both oligotrophic waters during a seasonal spawning event of little tunny (*Euthynnus alletteratus*) and in eutrophic waters where a seasonal upwelling event (lasting between May and September) gave rise to significant concentrations of zooplankton. Our survey thus enabled us to confirm that manta rays were likely consuming sergestid shrimp and calanoid copepods, as well as chaetognaths and fish eggs.

## Discussion

Effective establishment of marine protected areas for the conservation of species of concern depends on a robust understanding of their spatio-temporal distribution [24], [25], [26], [27]. Such understanding has now been gained for many marine species, including some that make basin-wide migrations [28], [29], [30], [31], [32], [33]. With technological improvements, the accuracy with which marine species can be localised has improved more than ten-fold [15], [34], [35] and a suite of ancillary data is now often collected as well as location to inform on migratory and foraging strategies [36], [37], [38].

Models of the spatio-temporal distribution of marine mega-vertebrates may enable both site- based conservation, such as the design and siting of marine protected areas, and the forecasting of climate change effects that may inform future mitigation measures (13). The Whale Shark Biosphere Reserve, declared in 2010, was intended to specifically enhance protection of whale sharks foraging off the Yucatan peninsula; however, it does not encompass the movements of manta rays tracked in this study [39], [40]. Further, it seems that manta ray aggregations coincide with some of the Caribbean's busiest shipping lanes [41], whose impact on manta rays is as yet unknown. Despite legal protection in Mexican waters (Norma Oficial Mexicana NOM-029-PESC-2006, 14 Feb 2007 Diario Oficial), occasional targeted and bycatch capture of manta rays still takes place (Anonymous Fishermen from Quintana Roo, Pers. Obs.) to be used for food and as bait in the shark fishery. There is also a growing demand in Asia for their gill rakers, which are used in traditional medicine [42]. The greatest impact on the aggregation in the next decade, however, may come from the region's expanding and largely unregulated marine megafauna tourism industry.

Acoustic tracking and photo-identification work have suggested strong site fidelity by manta rays to foraging areas in Indonesia [11], Hawaii [13] and Mozambique [43]. Our data add to this picture for the Atlantic Ocean; however, the capacity of manta rays for undertaking long-range migrations still remains uncertain. Without depth recording tags or detailed knowledge on the vertical structure of the water-column, we cannot confirm whether manta rays in this study, like many other planktivores, exhibited diel vertical migration, where animals track their diel migrating planktonic prey through the water column [44], [45]. Manta rays in this study likely foraged on three major prey types: (i) copepods (occurring in eutrophic waters), (ii) chaetognaths (known predators of copepods, influencing their distribution [46]) and (iii) fish eggs (spawned in oligotrophic waters where larval transport is optimised). However, manta 3, tagged in eutrophic waters (observed foraging on copepods), was re-sighted 57 days later foraging on fish spawn in oligotrophic waters, demonstrating that mantas can switch between habitat and prey types. Such plasticity in diet is worthy of further investigation.

Our data suggest that manta rays are foraging over large spatial scales (∼100 km long), too far offshore and too wide ranging to be included within existing MPA networks. Nevertheless, our data highlight significant site fidelity and association with frontal zones, which could be used to assess current biosphere reserve boundaries or to establish new dynamic protected areas overlaying the frontal region. The use of spatial data sets encompassing longer tracking periods are desirable to better inform manta ray management.

We provide a detailed description of the movements and environmental preferences of manta rays, highlighting what are likely foraging movements in shallow waters, in broad thermal fronts off an upwelling zone. We emphasise that few locations are received from protected areas and that manta rays may be subject to anthropogenic threats throughout their putative foraging range. While the broader migratory movements of manta rays are still not known, it is clear that satellite tracking technology has the potential to offer great inroads into understanding movements and contextualising spatially explicit threats to this species.
